# Expression of Topoisomerase II Alpha in Canine Colorectal Polyps, Adenomas and Adenocarcinomas

**DOI:** 10.1111/vco.70101

**Published:** 2026-08-12

**Authors:** Joanna Fiedorowicz, Katarzyna Paździor‐Czapula, Paweł Antoni Kołodziejski, Iwona Otrocka‐Domagała

**Affiliations:** ^1^ Department of Anatomic Pathology Faculty of Veterinary Medicine, University of Warmia and Mazury in Olsztyn Olsztyn Poland; ^2^ Department of Animal Physiology and Biochemistry Poznan University of Life Sciences Poznan Poland

**Keywords:** adenocarcinoma, colorectal, dog, ihc, topo IIα

## Abstract

With the development and increased accessibility of diagnostic methods in veterinary medicine, the possibilities for early detection of proliferative lesions in the canine large intestine have expanded over the past decade. Since in human medicine, topoisomerase II alpha (Topo IIα) is considered a prognostic and predictive marker in various tumours, including colorectal carcinoma, we aimed to verify the role of this enzyme in proliferative epithelial lesions of the canine large intestine. In this study, we examined and compared the expression of Topo IIα in normal rectal mucosa, hyperplastic polyps, adenomas, and adenocarcinomas of the large intestine in dogs. Each type of lesion included 20 cases, in which the expression of Topo IIα was evaluated using immunohistochemistry. The median percentage of Topo IIα positive cells was 29.4% (IQR 24.9–37.1) in polyps, 30.3% (IQR 24.6–34.1) in adenomas, 36% (IQR 26.2–42.0) in adenocarcinomas, and 7.8% (IQR 5.9–10.6) in normal intestinal mucosa. Statistical analysis using the Kruskal–Wallis test followed by Dunn's post hoc tests with Bonferroni correction revealed significantly higher Topo IIα expression in hyperplastic polyps (*p* = 0.013, *r* = 0.62), adenomas (*p* = 0.033, *r* = 0.57) and adenocarcinomas (*p* = 0.002, *r* = 0.73) compared with normal rectal mucosa, with large effect sizes. Our results may serve as a preliminary basis for the potential use of anthracyclines in the treatment of canine colorectal adenocarcinomas, as well as in multiple adenomas or polyps when surgical options are limited. This study presents new insights of Topo IIα expression in canine large intestine carcinogenesis and could provide a valuable foundation for further clinical research.

## 1 Introduction

The increased application of imaging techniques in veterinary medicine, including colonoscopy and computed tomography, has led to heightened detection of colorectal proliferative lesions in domestic animals, particularly in dogs [1, 2, 3]. In this group of canine lesions, epithelial neoplasms (adenomas and adenocarcinomas) and hyperplastic polyps are significantly predominant [3, 4]. A previous study showed that the most common proliferative colorectal lesion in dogs is adenocarcinoma, followed by adenoma and hyperplastic polyp, which is of particular concern given not only the prevalence of malignancy but also the potential risk of malignant transformation of adenomas [3].

It is noteworthy that chronic colitis in humans, including Inflammatory Bowel Disease (IBD), has been observed to heighten the risk of developing colorectal adenocarcinoma, as well as malignant transformation of adenomas or even non‐neoplastic polyps [5]. This is a relevant aspect in the context of the rising number of diagnosed cases of IBD in animals, particularly dogs, that have been observed in recent decades [6]. In consideration of the aforementioned factors, surveillance colonoscopy becomes a particularly valuable diagnostic tool for the early detection of hyperplastic lesions in the large intestine of canine patients.

Currently, research on the biology and malignant progression of proliferative epithelial colorectal lesions in dogs, as well as the search for new diagnostic, prognostic and predictive markers that would facilitate the development of targeted therapy, is gaining importance. This represents a trend that has undergone significant development in recent years within the domain of research on human colorectal adenocarcinoma [7, 8]. For many years, this neoplasm has been extensively studied due to its high prevalence and aggressiveness, making it one of the three most commonly diagnosed neoplasms in humans and one of the two most frequent causes of cancer‐related death [9]. Therefore, numerous diagnostic tools are sought for faster and more precise diagnosis. The aim of the research is also to search for markers of targeted therapy that would be helpful in precisely identifying neoplastic cells and adapting treatment to their specific characteristics.

Immunohistochemistry is a pivotal component of pathological evaluation, offering both cost‐effectiveness and expediency. Moreover, it facilitates the identification of potential predictive and prognostic markers. One of these markers, which is intensively evaluated in human colorectal adenocarcinoma and in many other neoplasia is topoisomerase IIα (Topo IIα). Topo IIα is a nuclear enzyme that plays an essential role in the cell cycle, influencing a number of processes including chromosome condensation, chromatid separation, DNA transcription, replication and repair [10, 11]. It has been established that the Topo IIα gene is expressed in both proliferating normal and transformed cells [12, 13]. In normal cells, Topo IIα expression is predominantly restricted to the S and G2/M phases of the cell cycle, which correspond to the phases of DNA replication and cell division. Concurrently, in transformed cells, particularly cancer cells, Topo IIα expression is elevated and less contingent on the proliferation rate of the cell [12]. Such dysregulation of Topo IIα expression can result from diverse mechanisms associated with cell transformation and proliferation and may significantly impact cancer development and treatment response. The significance of Topo IIα expression level in promoting cancer cell proliferation, survival, and metastasis, as well as its role as a predictive marker for response to anthracycline‐based chemotherapy, remains a subject of ongoing research. However, the findings of these studies are contentious [14]. It has been demonstrated that a high rate of recurrence, progression, and worse prognosis in human hepatocellular carcinoma, non‐muscle‐invasive bladder cancer, and lung carcinoma is associated with Topo IIα protein overexpression or gene amplification [14, 15, 16, 17]. Nevertheless, other studies have identified Topo IIα overexpression or amplification as a predictor of a favourable response to anthracycline‐based chemotherapy, which is indicative of a more positive prognosis, as observed in acute myeloid leukaemia [18, 19]. The results of studies on Topo IIα overexpression in human colon cancers appear to be particularly controversial. Some studies show that upregulation of Topo IIα can act as a negative prognostic or predictive factor [20, 21]. Conversely, other studies have demonstrated that high expression of this enzyme is linked to a beneficial patient outcome with colorectal adenocarcinoma [22, 23].

Topo IIα is the molecular target of anthracycline‐based chemotherapy due to its critical role in cell division [19]. Anthracyclines act as Topo IIα inhibitors, modulating the activity of this enzyme through two principal mechanisms: either by trapping the cleavage complex, thereby acting as a poison or toxin, or by inhibiting ATPase activity, leading to the arrest of enzyme rotation [24]. The expression of Topo IIα has been demonstrated to be positively correlated with the effectiveness of anthracycline‐based chemotherapy [25, 26]. Nevertheless, other studies indicate that Top IIα overexpression can result in resistance to topoisomerase inhibitors, due to the increased number of enzyme molecules available for targeting [27]. In view of the discrepancies, a significant objective of clinical research is to optimise the therapeutic efficacy of Top IIα‐targeting drugs, whilst concomitantly minimising the risk of toxicity [28].

The primary objective of this study was to evaluate the expression of Topo IIα in proliferative lesions in the canine large intestine and normal rectal mucosa. Furthermore, the second objective of the research was to compare the level of Topo IIα expression between hyperplastic polyps, adenomas, and adenocarcinomas to determine the possible role of this enzyme in carcinogenesis and tumour progression. The results of this study may provide insight into whether Topo IIα can be considered a potential marker for predicting colorectal neoplasms progression and assessing the efficacy of anthracycline‐based chemotherapy.

## 2 Materials and Methods

This study included 20 cases of each: hyperplastic polyps, adenomas, and adenocarcinomas from the canine large intestine (research groups). To compare our results with a normal canine large intestine, fresh samples of rectum were obtained from 4 dogs euthanized for reasons not related to gastrointestinal diseases (control group). Detailed information regarding age, sex, and breed of dogs was summarised in Table 1. All research groups samples constituted diagnostic specimens, submitted for routine histopathological examination to the Department of Anatomic Pathology, Faculty of Veterinary Medicine, University of Warmia and Mazury in Olsztyn. Samples were obtained through endoscopic procedures or excisional or incisional biopsy during surgery, fixed in 10% buffered formalin, processed routinely, embedded in paraffin, cut into 3 μm sections and stained with Mayer's haematoxylin and eosin (HE). Histologically, final diagnoses were established by consensus among three veterinary pathologists (J.F., K.P.‐C., I.O.‐D.) and classified according to the World Health Organization (WHO) International Histologic Classification of Tumours of Domestic Animals (Head et al. 2003) [29]. Immunohistochemical examination was performed manually and preceded by heat‐induced antigen retrieval in Tris‐EDTA buffer (pH 9.0) using PT‐Link (Dako, Glostrup, Denmark). The primary antibody was a mouse monoclonal antibody anti‐topoisomerase IIα (clone Ki‐S1, Dako), diluted 1:200. The visualisation system was based on the immunoperoxidase method, with 3,3‐diaminobenzidine (DAB) as a substrate (EnVision + System‐HRP, Mouse, Dako). The slides were counterstained with Mayer's haematoxylin. Positive (canine diffuse large B‐cell lymphoma; lymph node) and negative control slides were processed together with the evaluated slides. Microscopic assessment of Topo IIα expression was performed by the primary author (J.F.) using a light microscope (BX63, Olympus, Tokyo, Japan) with CellSens software (Olympus, Tokyo, Japan) and reviewed by two co‐authors (K.P.‐C., I.O.‐D.). The expression of Topo IIα was evaluated quantitatively in the areas of the slides with the most prominent expression (hotspots). The positive cells were counted manually (1000 cells/slide, 400× HPF) and the percentage of positive cells was calculated, based on the methodology used in previous studies in veterinary [30] and human medicine [22]. Only epithelial cells were counted, expression in the stroma was not considered. The positive expression of Topo IIα was recognised as nuclear, regular brown staining.

**TABLE 1:** Detailed information regarding colorectal polyps, adenomas, adenocarcinomas, and normal mucosa of the large intestine in dogs.

No.	Lesion	Age	Sex	Breed	Localization	Topo IIα + cells
1	P	5	f	French bulldog	Rectum	37
2	P	6	m	French bulldog	Rectum	30.5
3	P	2	m	French bulldog	Rectum	36.2
4	P	4	f	French bulldog	Rectum	27.3
5	P	11	m	Mongrel	Rectum	37.4
6	P	8	f	French bulldog	Large intestine	50.3
7	P	3	m	Mongrel	Rectum	21.6
8	P	10	m	West highland white terrier	Rectum	38.9
9	P	10	m	German shepherd	Rectum	22.7
10	P	7	m	Boston terrier	Rectum	31.7
11	P	2	f	Mongrel	Rectum	28.6
12	P	2	m	French bulldog	Colon	43.8
13	P	2	m	Welsh corgi	Rectum	38.2
14	P	1	f	French bulldog	Large intestine	25.6
15	P	ND	m	ND	Rectum	28.1
16	P	6	m	Mongrel	Rectum	26.4
17	P	4	m	French bulldog	Rectum	22.4
18	P	2	m	French bulldog	Rectum	21
19	P	3	m	Beagle	Rectum	30.2
20	P	2	m	Mongrel	Rectum	21.2
1	A	12	m	Mongrel	Rectum	17.9
2	A	6	m	Boxer	Rectum	26.1
3	A	6	m	Mongrel	Rectum	22.6
4	A	2	f	American bully	Rectum	27.1
5	A	12	f	French bulldog	Rectum	26.2
6	A	12	m	Yorkshire terrier	Rectum	31.5
7	A	5	f	French bulldog	Rectum	42.3
8	A	11	m	West highland white terrier	Large intestine	33.9
9	A	12	f	Mongrel	Rectum	30.6
10	A	5	m	ND	Colon	23.3
11	A	7	ND	ND	Large intestine	35.8
12	A	3	f	French bulldog	Large intestine	35.1
13	A	2	f	Bernese mountain dog	Large intestine	30.5
14	A	5	m	American pit bull terrier	Large intestine	34.8
15	A	8	f	Mongrel	Large intestine	31.9
16	A	ND	f	ND	Large intestine	47
17	A	6	f	ND	Large intestine	30.1
18	A	12	f	Golden retiriver	Large intestine	18.2
19	A	9	f	Dachshund	Large intestine	25
20	A	12	m	Havanese	Large intestine	19.8
1	AC	7	m	French bulldog	Rectum	21.9
2	AC	11	f	Mongrel	Rectum	39.1
3	AC	3	m	Mongrel	Rectum	42.1
4	AC	12	m	Labrador retriever	Rectum	56.5
5	AC	5	f	Bull terrier	Rectum	43.5
6	AC	13	m	Yorkshire terrier	Rectum	53.1
7	AC	7	m	Golden retriever	Rectum	30.2
8	AC	8	ND	ND	Large intestine	42
9	AC	7	m	Shih‐tzu	Large intestine	41.2
10	AC	10	f	Mongrel	Rectum	44.6
11	AC	10	m	ND	Rectum	36.7
12	AC	9	m	Mongrel	Rectum	38.1
13	AC	4	f	Bull terrier	Rectum	20.6
14	AC	13	m	Mongrel	Rectum	35.2
15	AC	7	m	Bull terrier	Rectum	28.2
16	AC	12	m	Nova scotia duck tolling	Rectum	22
17	AC	7	m	Great dane	Rectum	20.8
18	AC	10	m	Collie	Rectum	27.6
19	AC	11	m	West highland white terrier	Rectum	20.5
20	AC	13	m	Schnauzer	Rectum	29.8
1	N	7	m	Mongrel	Rectum	5.1
2	N	11	f	Mongrel	Rectum	6.2
3	N	3	f	Mongrel	Rectum	14.3
4	N	12	m	Boxer	Rectum	9.4

*Note:* The table summarises data regarding colorectal in dogs, including lesion (P‐polyp, A‐adenoma, AC‐adenocarcinoma, N‐normal), age (years), sex (f‐female, m‐male, ND – not determined), breed, localization and the percentage of Topo IIα‐positive cells [%].

Western blot study was performed using the protocol previously described by Leciejewska et al. in 2023 [31]. In brief, protein lysates from the canine colon and human colon cancer cell line LCC‐18 were isolated using RIPA buffer [32]. Next, 30 μg of total protein was separated by SDS‐PAGE using a 12% gel. Afterward, samples from the gel were transferred to PVDF membrane using Trans‐Blot Turbo System (Biorad, USA). The membranes were blocked with 3% BSA in TBS‐Tween‐20 (TBST) for 1 h at room temperature. After blocking, membranes were incubated overnight at 4°C with primary antibodies (dilution of 1:500). On the next day membranes were washed 3 times in TBST buffer and incubated with secondary antibody diluted 1:5000. Blot was visualised using Chemidoc Touch (Biorad, USA) and enhanced chemiluminescent reagent. The protein was normalised to β‐actin.

The expression of Topo II α between evaluated groups was compared using Kruskal–Wallis test, followed by a Dunn's test with Bonferroni correction. For pairwise comparisons, the effect sizes were calculated and expressed as r with adequate classification as small (≥ 0.1), medium (≥ 0.3), or large (≥ 0.5). The statistical analysis was performed using Statistica 13 software (Statsoft, Cracow, Poland), and differences were considered significant with *p* < 0.05.

## 3 Results

In canine colorectal hyperplastic polyps, the median percentage of epithelial cells immunopositive to Topo IIα was 29.4% (IQR 24.9–37.1) and ranged from 21% to 50.3%. In adenomas, the median percentage of epithelial cells expressing Topo IIα was 30.3% (IQR 24.6–34.1) and ranged from 17.9% to 47%. In adenocarcinomas, the median percentage of epithelial cells immunopositive to Topo IIα was 36% (IQR 26.2–42.0) and ranged from 20.8% to 56.5%. In the control group, the median percentage of epithelial cells expressing Topo IIα was 7.8% (IQR 5.9–10.6) and ranged from 5.1% to 14.4%. All data were collected and summarised in Table 1.

Interestingly, we observed that in normal mucosa (Figure 1) and in some polyps (Figure 2) Topo IIα expression was observed most often in crypts, whereas in adenomas (Figure 3) and adenocarcinomas (Figure 4), Topo IIα‐positive cells were distributed more diffusely and chaotically within the whole lesion. Additionally, in some cases of adenocarcinomas, Topo IIα expression was very prominent in multiple nucleoli.

**FIGURE 1:**
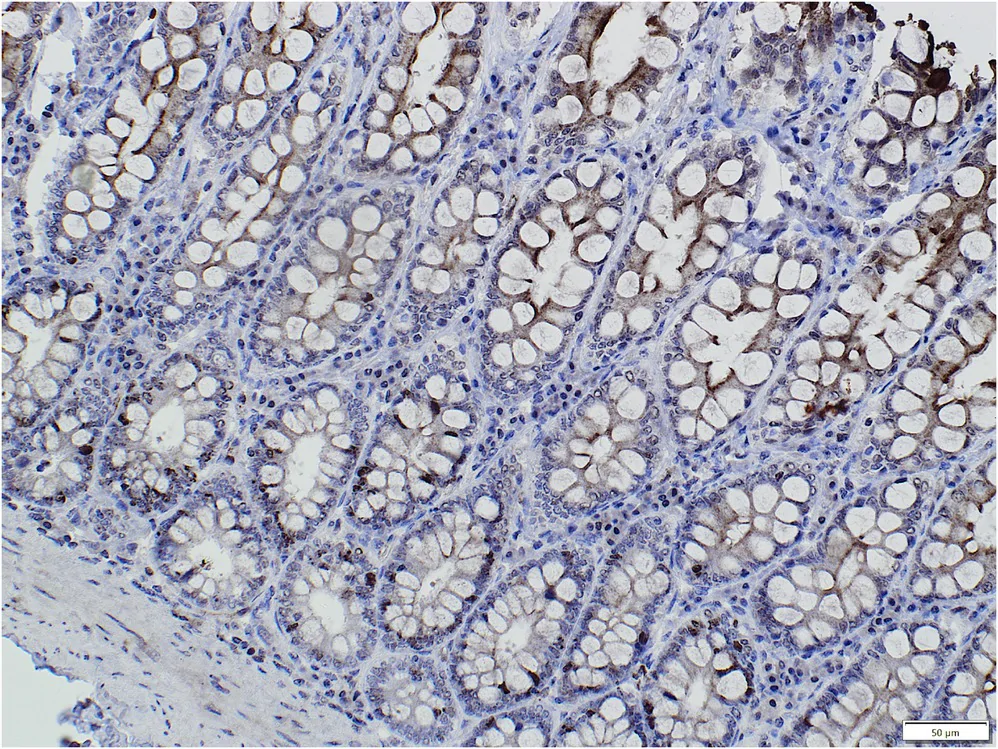
Minimal expression of Topo IIα in normal mucosa is present predominantly in the lower crypts.

**FIGURE 2:**
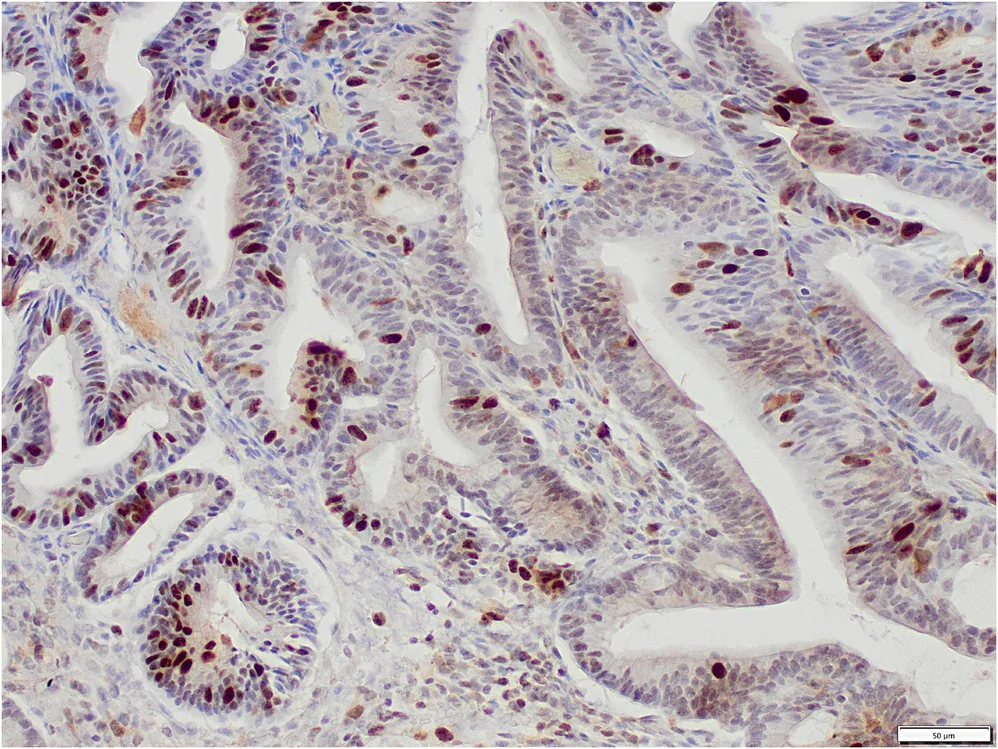
Expression of Topo IIα in polyps is present mostly in the crypts, with a predominance of basal expression.

**FIGURE 3:**
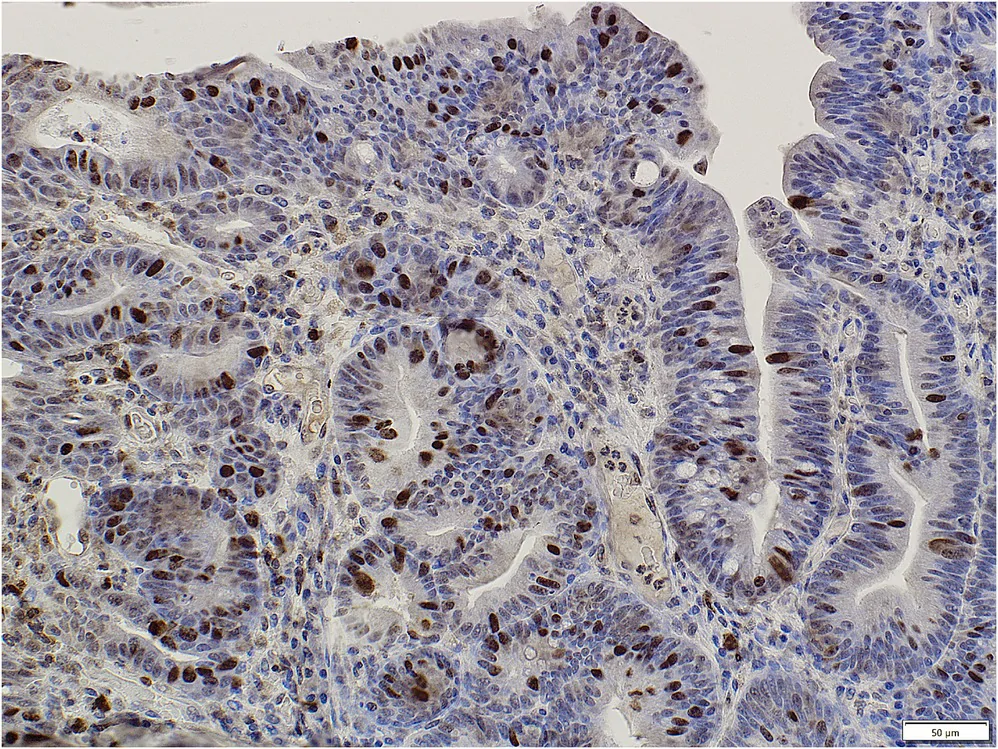
Expression of Topo IIα in adenoma; positive cells in double and multilayered crypts are sometimes observed on different levels, presenting more diffuse pattern.

**FIGURE 4:**
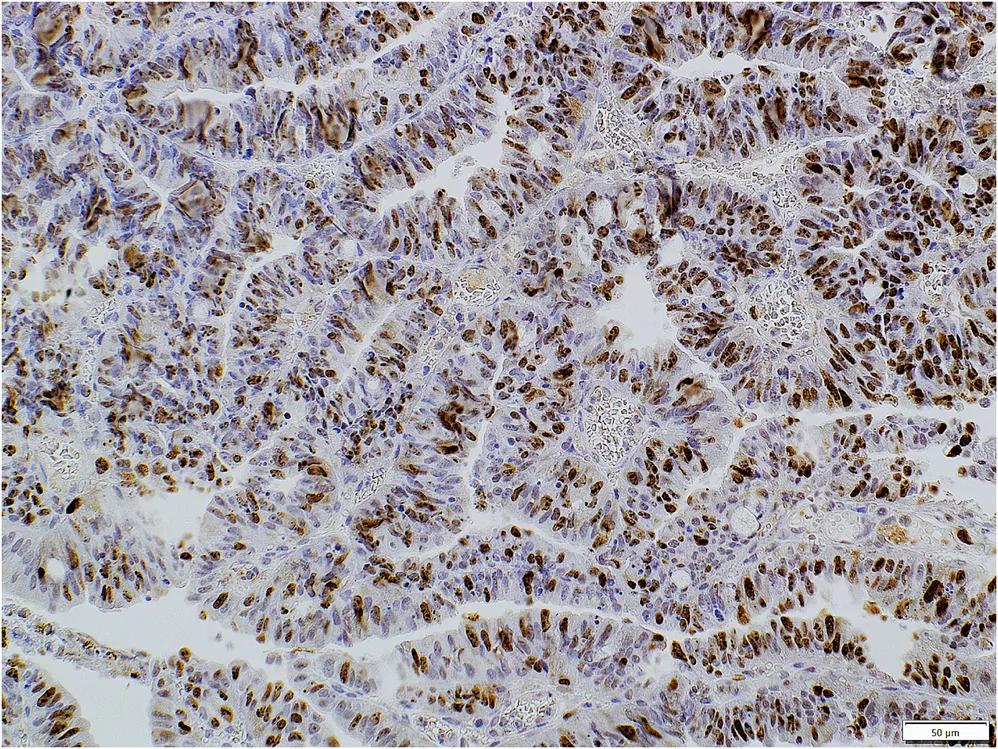
Diffuse and chaotic expression of Topo IIα present in adenocarcinoma. Tumour cells forming multilayers with positive cells at different levels.

To further assess antibody reactivity, Western blot analysis was performed using protein lysates derived from canine colon tissue and the human colon cancer cell line LCC‐18. The expected molecular weight of full‐length Topoisomerase IIα is approximately 170 kDa. In both human and canine samples, the immunoreactive band was detected at approximately 170 kDa, corresponding to the expected molecular weight of Topo IIα. Additional lower‐molecular‐weight bands were also observed in both species (Figure S1). These bands may represent degradation products, alternatively processed protein forms, post‐translationally modified variants, or non‐specific antibody binding. This pattern is also consistent with standard Western blot sample preparation, including protein denaturation and disulfide bond cleavage, which is known to cause additional bands for several anti‐topoisomerase II antibodies. This phenomenon has also been reported by other anti‐topoisomerase II antibodies. Therefore, the observed Western blot pattern suggests reactivity consistent with previously published observations and manufacturer‐reported data for anti‐Topo IIα antibodies but does not definitively confirm antibody specificity. Furthermore, the Western blot findings do not rule out the possibility of non‐specific binding. We acknowledge that more rigorous validation approaches, such as peptide‐blocking experiments, knockdown/knockout controls, or mass spectrometry‐based protein identification, would provide stronger evidence of antibody specificity.

Statistical analysis revealed that Topo IIα expression in hyperplastic polyps (*p* = 0.013, *r* = 0.62), adenomas (*p* = 0.033, *r* = 0.57), and adenocarcinomas (*p* = 0.002, *r* = 0.73) was significantly higher, with large effect sizes, compared to normal rectal mucosa. However, there was no statistically significant difference in Topo IIα expression between hyperplastic polyps, adenomas, and adenocarcinomas. The percentage of Topo IIα‐positive cells in each of the evaluated groups was presented in graphs (Figure 5).

**FIGURE 5:**
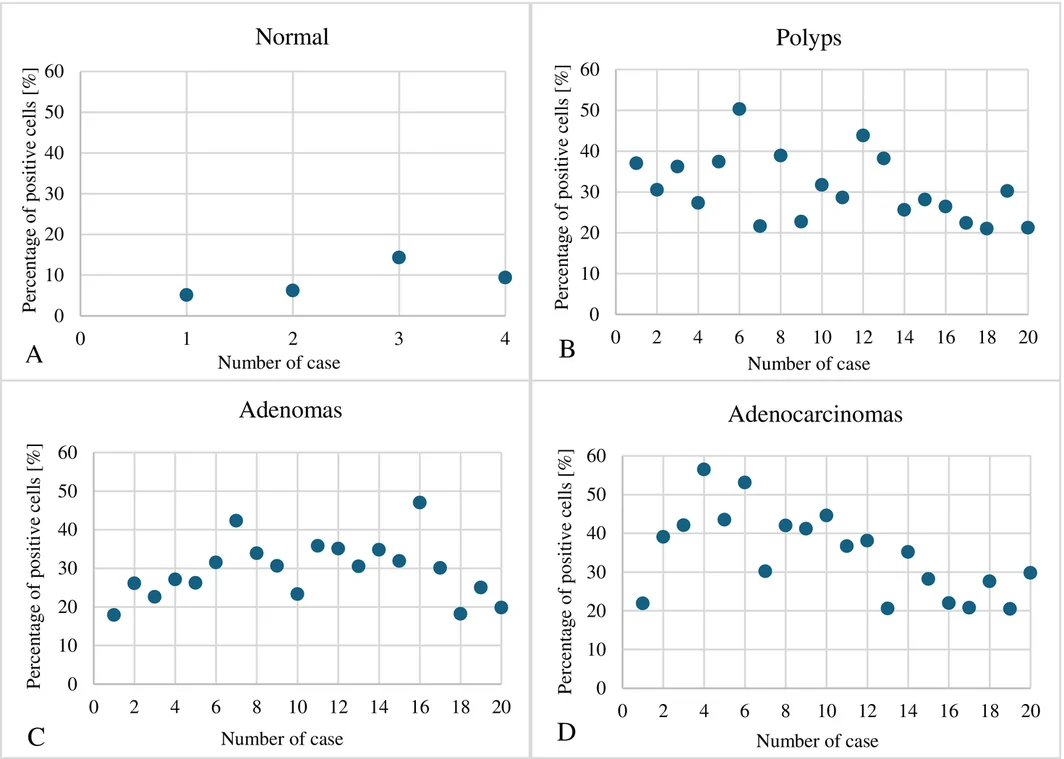
Distribution of Topo IIα positive cells in percentage in (A) normal large intestine, (B) polyps, (C) adenomas and (D) adenocarcinomas.

## 4 Discussion

In the treatment of canine colorectal proliferative lesions, wide, complete surgical resection is the most commonly recommended intervention [33]. However, in the case of malignant neoplasms deeply infiltrating the intestinal wall and incompletely removed, the use of adjuvant chemotherapy would be indicated. Few studies are available on the use of various cytostatic‐based adjuvant chemotherapy protocols for treating colorectal adenocarcinoma in dogs [34, 35]. These studies have promising results and clearly demonstrate the benefits of this therapeutic approach, including keeping patients in clinical remission and extending their survival [34, 35]. Therefore, it is advisable to search for novel prognostic tools and factors that could be targeted in the individual chemotherapy of colorectal adenocarcinoma in dogs. One of the candidate markers with prognostic and predictive properties is Topo IIα.

In consideration of the above‐mentioned observations, the objective of the present study was to evaluate the expression of Topo IIα in colorectal proliferative lesions in dogs. Our findings revealed that Topo IIα is not only overexpressed in canine adenocarcinomas, but also in hyperplastic polyps and adenomas when compared with normal rectal mucosa. This is the first study to demonstrate this phenomenon. These findings justify future investigation of Topo IIα as a potential biomarker of response to anthracycline‐based chemotherapy. The proposed treatment could be effective for infiltrating and metastatic adenocarcinomas, as well as multiple adenomas or polyps when surgical removal is challenging or impossible. Nevertheless, this hypothesis must be confirmed in future clinical studies.

In humans colorectal adenocarcinoma, overexpression of Topo IIα was evaluated in large cohort studies and has been shown to have prognostic and predictive value [20, 22, 23]. Nonetheless, the results of these studies have been found to be somewhat divergent. Gao et al. (2012) showed that, in the majority of colorectal adenocarcinomas examined, 31%–70% of tumour cells expressed Topo IIα, which was defined as a moderate expression, while according to Coss et al. (2009), expression greater than or equal to 20% was classified as moderate to high. Hendricks et al. (2019) reported comparable levels of Topo IIα expression in colon cancer cells, with a mean and median expression rate of 52% and 53.8%, respectively. In the present study, the median percentage of immunopositive cells to Topo IIα in adenocarcinomas was determined to be 36%, with a range from 20.8% to 56.5%. Consequently, our study has demonstrated that canine colorectal carcinoma cells overexpress Topo IIα at a level comparable to human colorectal adenocarcinomas.

Surprisingly, in our study, the median percentage of Topo IIα‐positive epithelial cells was quite similar in all examined colorectal proliferative lesions. The median Topo IIα expression in hyperplastic polyps was 29.4%, ranging from 21% to 50.3%, while in adenomas it was 30.3%, ranging from 17.9% to 47%. As the Topo IIα is considered a proliferative marker in several human neoplasms [36, 37, 38], including colorectal adenocarcinoma [39], we expected that the expression of Topo IIα would be significantly higher in neoplastic versus non‐neoplastic and malignant versus benign epithelial lesions. In humans, expression of Topo IIα is higher in malignant than in benign tumours of various types [39, 40, 41, 42]. Furthermore, we demonstrated high variability of Topo IIα expression within each of the research groups, and both the overall range and interquartile range were highest in adenocarcinomas. This variability has been shown in human colorectal adenocarcinoma, where the expression of Topo IIα ranged from 0% to 100% [20, 22, 23]. In humans, expression of Topo IIα in normal rectal epithelium shows circadian variation [43], but is not known whether similar variation also occurs in colorectal neoplasms.

The overexpression of Topo IIα in hyperplastic polyps and adenomas, as observed in our study, may indicate the potential risk of malignant transformation of these lesions. Increased expression of this enzyme was observed in dysplastic lesions and adenomas of the human colon and was correlated with the level of dysplasia [40]. However, the same researchers did not observe any differences in Topo IIα expression between well‐differentiated and poorly differentiated carcinoma.

In canine veterinary oncology, expression of Topo IIα has been evaluated in several tumour types, including lymphomas [44, 45], mammary tumours [29] and cortisol‐secreting adrenocortical tumours, but only in the latter this expression associated with poor prognosis [46]. In human colorectal adenocarcinoma, the prognostic value of Topo IIα expression has been evaluated, but the results are contradictory. Several studies have demonstrated that higher expression of Topo IIα was associated with better clinical outcomes and longer survival [22, 23]. Based on those results, validation of the expression level in selected dogs with colorectal lesions before application of anthracyclines could provide useful information about expected treatment response. However, this hypothesis should be confirmed with further research. In contrast, another study has shown that higher expression of this marker was correlated with the more advanced tumour stage and resistance to Topo IIα inhibitors therapy [20]. Since the Topo IIα expression in canine colorectal proliferative lesions in the present study was at similar levels, regardless of the lesion type, the potential prognostic and predictive significance of this marker necessitates further investigation, encompassing a more substantial cohort of cases and incorporating clinical studies.

We showed that expression of Topo IIα in epithelial cells of normal rectal mucosa was minimal, with levels below 10%. A comparable survey conducted in humans revealed minimal expression of Topo IIα in normal large intestine mucosa, associated with the normal proliferation of epithelial cells in lower crypt zones [47]. Furthermore, we demonstrated that the expression of Topo IIα in hyperplastic polyps, similarly to normal mucosa, was observed most frequently in crypts, whereas in adenomas and adenocarcinomas, the distribution of Topo IIα‐positive cells was diffuse, which is comparable with a previous study in humans [47]. This finding probably reflects the loss of differentiation of epithelial cells during tumorigenesis.

This study has some limitations. Our study was based only on the immunohistochemistry method, and the quantitative analysis was performed manually by a single pathologist under supervision; therefore, some subjectivity and imprecision probably could not be avoided, especially since formal reproducibility assessment was not performed. For that reason, more accurate quantitative molecular methods, such as quantitative real‐time PCR (qRT‐PCR) or fluorescence in situ hybridization (FISH), as well as reproducibility assessment, should be applied in future studies. Furthermore, reproducibility between hotspot evaluations should also be assessed, as it was not formally quantified. In addition, hotspot‐based methodology may overestimate expression. However, we selected this method because Topo IIα is considered a proliferation marker, and we aimed to compare the maximal differences in expression between hyperplastic and neoplastic lesions. Thus, in future studies, especially when evaluating only one lesion type, comparison between hotspot‐based and random‐area expression assessment could be considered.

In summary, our data demonstrate, for the first time, that Topo IIα is not only overexpressed in canine adenocarcinomas, but also in hyperplastic polyps and adenomas. The median level of this expression was found to be similar in all examined colorectal proliferative lesions; however, much higher than that observed in normal mucosa. These results may provide novel insights into the phenomenon of carcinogenesis and tumour progression in the colon environment in dogs. Although the prognostic value of Topo IIα seems to be questionable, the distinct upregulation of its expression in all proliferative lesions of the large intestine is a good basis for future studies on anthracycline implementation in the therapeutic protocols.

## Funding

This work was funded by the Minister of Science under the Regional Initiative of Excellence Program.

## Ethics Statement

The study used only archival diagnostic specimens collected during routine veterinary procedures; therefore, ethical approval was not required in accordance with institutional and national regulations. All collected data were anonymized, and written informed consent was obtained from the animal owners.

## Conflicts of Interest

The authors declare no conflicts of interest.

## Supporting information


**Figure S1:** demonstrating Western blot analysis of Topoisomerase IIα in normal canine colon tissue and LCC‐18 cells. A main band was detected at approximately 170 kDa, with additional lower‐molecular‐weight bands observed in both species. β‐actin (~42 kDa) served as a loading control.

## Data Availability

The Western blot data supporting the findings of this study are provided as Supporting Information. Additionally, data that support the findings of this study are available from the corresponding author upon reasonable request.
