# Supplementary material for: Expression of Topoisomerase II Alpha in Canine Colorectal Polyps, Adenomas and Adenocarcinomas
Source: Vet Comp Oncol. 2026 Aug 12;24(4):781–9. doi: 10.1111/vco.70101 (PMC13745624; doi:10.1111/vco.70101)
Supplement: Supplementary file 1 [file VCO-24-781-s001.docx]

We would like to confirm that the cross-species validation of the antibody used in our study was performed according to the recommendations of Ramos-Vara et al. (J Vet Diagn Invest 2008;20:93). Specifically, we validated the antibody using Western blot analysis and demonstrated its reactivity with antigens of the appropriate molecular weight.

The antibody demonstrated a comparable binding pattern in canine tissue (protein lysates

collected from the canine colon) and in the human colon cancer cell line LCC-18 (Lundqvist M, Mark J, Funa K, et al. Characterization of a cell line (LCC-18) from a cultured human neuroendocrine-differentiated colonic carcinoma. European Journal of Cancer and Clinical Oncology, 27(12), 1663-1668; <https://www.cellosaurus.org/CVCL_W648>), which expresses the human form of the target protein for which the antibody was originally developed. This confirms that the antibody specifically recognizes the protein of interest in both human and canine samples.

Because this antibody was designed primarily for immunohistochemistry and immunofluorescence, its Western blot results show multiple bands, a common finding for antibodies detected in IHC/IF studies. This pattern is consistent with standard Western blot sample preparation, including protein denaturation and disulfide bond cleavage, which is known to cause additional bands for several anti-topoisomerase II antibodies. This phenomenon has also been reported by other anti-topoisomerase II antibody manufacturers (e.g., ThermoFisher Scientific; catalog number 7153-MSM2-P1 - <https://www.thermofisher.com/antibody/product/Topoisomerase-II-alpha-Antibody-clone-TOP2A-1362-Monoclonal/7153-MSM2-P1> and <https://www.neobiotechnologies.com/product/topoisomerase-ii-alpha-proliferation-drug-resistance-marker-7/?srsltid=AfmBOorPWZEUE_FEESBWM4Aidaj_hEhi-RIIHvXsmRSQVuZXw3990nTB>).

In our study, we used protein lysates prepared in RIPA buffer supplemented with Laemmli buffer, resulting in completely reduced and denatured protein samples. This explains the appearance of several bands in the Western blot, which is typical for this type of antibody under reducing conditions.

We acknowledge that other validation strategies—such as the use of a blocking peptide—could further confirm antibody specificity. However, for this particular antibody, the manufacturer does not provide a blocking peptide or disclose the sequence of the immunizing antigen, making this method unavailable.

Western blot method was performed as we previously described : Leciejewska, N., Pruszyńska–Oszmałek, E., Nogowski, L., Sassek, M., Strowski, M. Z., & Kołodziejski, P. A. (2023). Sex-specific cytotoxicity of ostarine in cardiomyocytes. *Molecular and Cellular Endocrinology*, *577*, 112037.

**Supplementary figure 1**.

Supplementary figure 1 demonstrating Western blot analysis of Topoisomerase IIα in normal canine colon tissue and LCC-18 cells. A main band was detected at approximately 170 kDa, with additional lower-molecular-weight bands observed in both species. β-actin (~42 kDa) served as a loading control.
